# Dog demography and ecology with reference to rabies in the Amhara region, Ethiopia

**DOI:** 10.1016/j.heliyon.2024.e41582

**Published:** 2024-12-31

**Authors:** Liuel Yizengaw Adnie, Wudu Temesgen Jemberu, Adane Bahiru Woreta, Adugna Berju, Araya Mengistu, Zeleke Tesema Wondie, Wassie Molla, Sefinew Alemu Mekonnen

**Affiliations:** a Department of Veterinary Laboratory Technology, College of Agriculture and Natural Resource, Debre Markos University, P O Box 269, Debre Markos, Ethiopia; b Department of Veterinary Epidemiology and Public Health, College of Veterinary Medicine and Animal Sciences, University of Gondar, P. O. Box 196, Gondar, Ethiopia; c Sirinka Agricultural Research Center, P. O. Box 74, Woldia, Ethiopia; d International Livestock Research Institute, Addis Ababa, Ethiopia; e Sekota Dry-land Agricultural Research Center, Sekota, Ethiopia

**Keywords:** Amhara, Demography, Dogs, Ecology, Ethiopia, Rabies

## Abstract

Knowledge of domestic dog ecology and demography has been recognized as central to the design of an effective rabies control program. The study was conducted to assess owned dogs' ecology and demography and to identify predictors associated with dog ownership and rabies occurrence in the Amhara region, Ethiopia.

**Method:**

ology: The study employed dog census and questionnaire surveys of 907 households selected using a multistage sampling technique from six rural and six urban districts of the Amhara region, Ethiopia. The ecology and demography of owned dogs in the selected areas were recorded and described using descriptive statistics. Mixed-effect logistic regression models were used to identify factors associated with dog ownership and rabies occurrence.

**Results:**

A total of 6609 dogs were estimated from 42 kebeles in the 12 study districts. The male-to-female ratio of dogs was 1.7:1.0, and the mean age of dogs was 3.2 years. The proportion of households who owned at least one dog was 5.9 %. The average number of dogs per dog-owned household was 1.3. Dog to household ratio was 1.0:13.0, and dog to human ratio was 1.0:48.5. The majority of the dog owners (97 %) keep dogs for home guard and livestock herding. Only 57 % of the dogs were confined, and 16 % of them were vaccinated. Ninety-one percent of the dog owners did not practice neutering and spaying for dog population control. Religion, livestock ownership pattern, and occupation were associated with dog ownership (p < 0.05). Community residence and age of respondents were associated with rabies occurrence (p < 0.05), while zone was associated with both dog ownership and rabies occurrence at p-value <0.05.

**Conclusions:**

The study indicated a low dog population relative to humans, which might make dog-based rabies control manageable. But at the same time, most dogs were not properly managed (confined and vaccinated), which calls for more awareness about responsible dog ownership to reduce zoonotic disease risk, including rabies. Generally, the study provides useful information about the demography and ecology of owned dogs in relation to rabies for making proper and effective rabies control strategies and is important to design the spatial distribution of rabies vaccination in dogs. However, as the study did not include stray dogs, which have great contributions to the spread of rabies, the information should be used with this caveat into consideration.

## 1 Background

In many places of the world, dogs (Canis familiaris) are the most common and plentiful carnivore animals. Dogs are extremely reliant on humans or human activity [1]. The useful attributes of dogs include the ability to hunt cooperatively, guard and defend people, livestock, and other property; pull vehicles and carry goods; serve as objects of barter; and act as social partners and companions [2]. In Ethiopia, most households own dogs, usually for guarding property. However, the practice of keeping dogs is not without problems. In nearly all parts of the world, dogs pose serious human health, socioeconomic, and animal welfare problems [3]. Dog’ populations may rapidly grow to such an extent that the health risks for humans become serious and the environment begins to suffer considerably if responsible dog ownership and management are not in place [3].

Rabies is a neglected zoonotic disease that affects all warm-blooded animals and kills about 59,000–60,000 people per year, with the majority of cases in Asia (60 %) and Africa (36 %) [4,5]. The broad range of reservoir animals and environments in which rabies virus is found creates a complex ecologic and epidemiologic dynamic that intricately links the health of humans, animals, and the environment [4]. Globally, 20 million people are vaccinated annually as a result of rabies exposures [4]. It also causes about 3.7 million disability-adjusted life years (DALYs) and economic losses of 8.6 billion US$ per year [4]. The annual costs of rabies in Africa and Asia were estimated at US$ 583.5 million, most of which were due to costs of post-exposure prophylaxis (PEP) [6]. In rabies-endemic countries, it also has significant economic importance due to its effect on livestock. In Africa and Asia, the annual costs of livestock losses as a result of rabies were estimated to be US$ 12.3 million [6].

In Ethiopia, the first outbreak of rabies in dogs was reported in 1884, particularly in the former province of Tigray, Gondar, Gojjam, and Wollo, and later in Addis Ababa [7]. Currently, human rabies is an immediately reportable disease. A surveillance data compiled in 2007 indicated 15,178 (3.4/100,000 populations) exposures to rabies and 272 fatal cases, with more than 88 % of the exposure being due to dog bites [8]. A retrospective study between 2001 and 2009 showed 386 fatal human cases with an annual range of 35–58 deaths [8]. The same surveillance data indicated that the highest incidence was registered in the Amhara region [8]. A prospective study done at the North Gondar zone of the Amhara region indicated an annual rabies incidence of 2.33 cases per 100,000 humans; 412.83 cases per 100,000 dogs; 19.89 cases per 100,000 cattle; 67.68 cases per 100,000 equines; and 14.45 cases per 100,000 goats [9].

About 99 % of the human rabies cases that occur in developing countries are due to dog bites [[9], [10], [11], [12]]. Similarly, domestic dogs are the principal reservoirs and sources of infection for humans and livestock rabies in Ethiopia [6,9,13]. Dog rabies vaccination is the primary component of a rabies control program; however, reaching the recommended 70%–80 % vaccination levels in dogs can be challenging in resource-limited countries [14]. In most of the developing countries with poor infrastructure and inadequate resources, the major constraints to effective rabies control in dogs are economic and logistical rather than technical problems [15].

In Ethiopia, although there are no formal studies, it is estimated that there is one owned dog per five households [16]. This high population with abundant stray dogs and poor management of owned dogs and the limited vaccination practice contributes to the high endemicity of canine rabies. There is no official rabies control program enforced yet, while dog vaccination could reduce human deaths attributable to rabies [17]. The vaccination coverage in the country is about 20 % in urban areas and non-existent in rural areas [18,19]. In addition to logistic and economic problems in using mass dog vaccination, lack of information on dog ecology and demography is one of the main challenges for planning control of dog-mediated rabies [19].

Rabies epidemiology in the dog reservoir is directly associated with dog demography (the study of populations and is concerned with the size, the age and sex composition of the population, and how the population changes over time) and dog ecology (involves studies on dog population density, dog population structure, and pattern of dog ownership); thus, better understanding of dog demography and ecology is useful for designing appropriate rabies control measures in the dog population [20,21]. Knowledge of dog demography and ecology is important to estimate realistic vaccination coverage that disrupts rabies transmission in the long term. It is also important to understand the human-to-dog ratio because it helps to estimate the level of contact between dogs and humans. Moreover, identifying factors associated with the occurrence of rabies is relevant for control intervention. Like many parts of Ethiopia, in the Amhara region, there is a lack of well-organized data on dog demography and ecology. Therefore, this study was conducted with the objectives of describing and characterizing owned dog demography and ecology and identifying factors associated with dog ownership and rabies outbreaks in the Amhara region, Ethiopia.

## 2 Materials and methods

### 2.1 Study area and study population

**Fig. 1:**
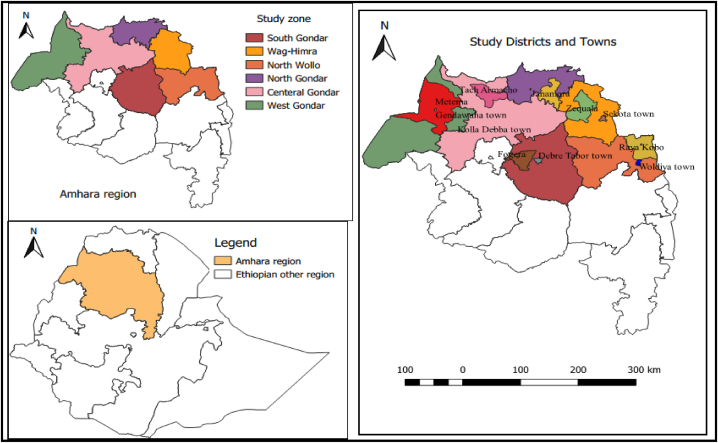
Map of the study area showing study zones, districts, and towns in the Amhara region, Ethiopia.

The study was done in the Amhara region of Ethiopia, which is located between 9° 20 and 14° 20 and 36**°** 20 and 40**°** 20 latitude and longitude, respectively. This region was selected based on the lack of well-organized dog ecology and demography data, along with the high incidence rabies outbreak and the intention of the project during that specified period of time. It covers approximately an estimated area of 154,709 km^2^. The Amhara region is divided into 13 administrative zones, namely West Gondar, Central Gondar, North Gondar, South Gondar, West Gojjam, Bahirdar special zone, Awi, East Gojjam, Wag-Himra, North Wollo, South Wollo, Oromia special zone, and North Shoa. In the region, about 22,876,991 human populations live in 3,983,768 households, which results in an average of 5.7 persons to a household. The region has an estimated density of 147.9 people per square kilometer [22]. About 88 % of the population lives in rural areas [22]. Twelve districts 6 rural districts (areas that are located outside principal towns of the zone) and 6 towns (principal towns of the zone) from six administrative zones of the region were included in the study: Tach Armachiho district and Koladiba town from Central Gondar zone, Janamora district and Debark town from North Gondar zone, Fogera district and Debretabor town from South Gondar zone, Metema district and Gendawuha town from West Gondar zone, Raya Kobo district and Woldia town from North Wollo zone, and Ziquala district and Sekota town from Wag-Himra zone (Fig. 1). The study populations were owned dogs and the households residing in the 12 selected districts.

### 2.2 Study design and data collection

The study involved a cross-sectional dog census and sample questionnaire survey of households between December 2020 and June 2021. The dog census was done by trained enumerators from residents of the selected kebeles (the smallest administrative unit) within the study districts. The enumerators traveled systematically by villages and streets and reached out to all households in the kebele and recorded the number of dogs and dog demographic data. In parallel, a sample of households was selected for the questionnaire survey by simple random method and interviewed to collect data related to participant socio-demographics associated with dog demography and ecology and rabies outbreak experience in the previous five years. Prior to the data collection, selected respondents (household heads) were checked for awareness of rabies based on rabies clinical case definition. The questionnaire consisted of three sections. The first part contained 25 questions on participant household socio-demographic characteristics, dog ownership, and rabies outbreaks. The second part was related to information on dog demography and population characteristics (25 questions). The third part of the questionnaire was referring to dog management and dog handling practices (31 questions) (Appendix 1). The questionnaire was originally prepared in English, and then it was translated to the local language, Amharic, and then back to English by an external translator to check for consistency. The Amharic version of the questionnaire was administered by face-to-face interviews.

Sampling methods and sample size determination.

A multistage sampling method was employed to select the study participants. First, the administrative zones were selected purposively based on ease of accessibility and the interest of the university project mandate area associated with a high rabies outbreak history in those areas. Then, for the dog census study, districts within zones and kebeles within districts, and for the household sample survey, villages within the kebeles and households within villages, were selected simply randomly. A list of kebeles was obtained from the district agricultural office, and a list of villages and households was obtained from the kebele administrative offices. For the purpose of dog population estimation, all dog-owned households within kebeles were included, and all available owned dogs were counted (Appendix 3). But for dog ownership and occurrence of rabies outbreak study, households were selected randomly without considering dog ownership using a computer-based random number. Within households, the heads of the households were selected for interview. However, a member of the household was substituted when the head of the household was not available.

The required sample size for the household survey was determined using the Cochran formula for categorical data [23], n = $\frac{\;z^{2}\;p\left(1-p\right)}{d^{2}}$, where n = required sample size, p = expected proportion of dog ownership and rabies outbreak experience history in the study population, z = the value for a selected alpha level, and d = margin of sampling error. Using a 50 % expected proportion, a 95 % confidence interval, and a 5 % desired absolute precision, the sample size was calculated for the rural and urban households separately. Based on the formula, the total number of households required for both urban and rural districts was 768 (384 for each). But, finally, the total sample size was increased to 907 households (453 for rural and 454 for urban areas) to maximize precision. The sample size was distributed among the administrative zones proportional to population size.

### 2.3 Data management and analysis

Descriptive statistics were applied for summarizing socio-demographic characteristics of study participants, dog demography, and ecology and management data. The number of dogs per district was estimated by multiplying the average number of dogs in the sampled households with the number of households in a district. The average number of dogs in the sampled households was estimated by dividing the number of dogs counted by the number of households covered by the census. Similarly, the average number of dogs per human was estimated by dividing the number of dogs counted by the total number of humans from all households covered by the census. Dog density was estimated by dividing the number of dogs counted in the districts by the total area of the districts. The human population data of the districts used in the calculation was obtained from the Central Statistical Agency of Ethiopia [22].

Mixed-effect logistic regression models were used to identify factors associated with dog ownership and rabies outbreaks. Sex, age, residence, zone, family size, religion, occupation, educational status, marital status, income level, duration of years lived in the area, dog, cat, and livestock ownership, and awareness of rabies were tested as covariates. Factors with a likelihood-ratio test P-value of 0.25 in univariable mixed-effect logistic regression were considered for entry into multivariable mixed-effect logistic regression models. Variables significantly associated in the univariable analysis were tested for multicollinearity. When two variables had a correlation coefficient of ≥0.7, only one of the variables was included in the further multivariable analysis. Predictor variables with P < 0.05 were retained in the final multivariable mixed-effect logistic regression model. Confounding was checked during the model-building process by evaluating the change in the beta estimate of other variables when a variable was removed from the model. If this change in beta estimate was >30 %, the variable was considered a confounder [23]. All two-way interactions between variables in the final multivariable models were tested. Model validation was done using the Hosmer and Lemeshow test [24]. The statistical analysis was done using STATA version 16 (Stata Corp., College Station, Texas, 77845, USA).

## 3 Results

### 3.1 Socio-demographic characteristics of respondents

The distribution of the 907 household questionnaire survey respondents in the six study zones was 161 (17.8 %), 154 (17 %), 151 (16.6 %), 148 (16.3 %), 152 (16.8 %), and 141 (15.5 %), respectively, in the Central Gondar, North Gondar, South Gondar, West Gondar, North Wollo, and Wag-Himira zones. The detailed socio-demographic information of the household questionnaire survey respondents is presented in Table 1.

**Table 1:** Socio-demographic structure of respondents (N = 907) in rural and urban districts of the Amhara region, Ethiopia.

Variable	Category	Respondents		
		Urban (%)	Rural (%)	Total (%)
Sex	Male	266 (58.6)	311 (68.7)	577 (63.6)
	Female	188 (41.4)	142 (31.3)	330 (36.4)
Age	Young (<18 years)	35 (7.7)	18 (4.0)	53 (5.8)
	Youth (18–30 years)	140 (38.8)	131 (28.9)	271 (29.9)
	Adult (>30–60 years)	204 (44.9)	232 (51.8)	436 (48.1)
	Old (>60 years)	75 (16.5)	72 (15.9)	147 (16.2)
Educational level	College and above	122 (26.9)	20 (4.4)	142 (15.7)
	Secondary	126 (27.8)	52 (11.5)	178 (19.6)
	Primary	128 (28.2)	159 (35.1)	287 (31.6)
	Illiterate	78 (17.2)	222 (49)	300 (33.1)
Religion	Orthodox Christian	428 (94.3)	422 (93.2)	850 (93.7)
	Muslim and other	26 (5.7)	31 (6.8)	57 (6.3)
Income	Low income (<1500 ETB)	72 (15.9)	70 (15.5)	142 (15.7)
	Medium income (1500–4000 ETB)	129 (28.4)	117 (25.8)	246 (27.1)
	High income (>4000 ETB)	35 (7.7)	14 (3.1)	49 (5.4)
	Not known	218 (48)	252 (55.6)	470 (51.8)
Occupation	Farmer	59 (13.)	358 (79)	417 (46.0)
	Business	102 (22.5)	52 (11.5)	154 (17.0)
	Government Employee	125 (27.5)	19 (4.2)	144 (15.9)
	Private/self-employed	95 (20.9)	13 (2.9)	108 (11.9)
	Others	73 (16.1)	11 (2.4)	84 (9.3)
Marital status	Divorced	36 (7.9)	22 (4.9)	58 (6.4)
	Married	265 (58.4)	361 (79.7)	626 (69)
	Single	133 (29.3)	56 (12.4)	189 (20.8)
	Widowed	20 (4.4)	14 (3.1)	34 (3.7)
Family size	Small (<3 persons)	99 (21.8)	52 (11.5)	151 (16.6)
	Medium (3–6 persons)	250 (55.1)	224 (49.4)	474 (52.3)
	Large (>6 persons)	105 (23.1)	177 (39.1)	282 (31.1)
How long in the area lived	<5 years	78 (17.2)	68 (15)	146 (16.1)
	5–10 years	61 (13.4)	55 (12.1)	116 (12.8)
	>10–20 years	113 (24.9)	93 (20.5)	206 (22.7)
	>20–30 years	90 (19.8)	102 (22.5)	192 (21.2)
	>30 years	112 (24.7)	135 (29.8)	247 (27.2)
Livestock ownership	Yes	150 (33.1)	345 (76.2)	495 (54.6)
	No	304 (66.9)	108 (23.8)	412 (45.4)
Cat ownership	Yes	209 (46)	210 (46.4)	419 (46.2)
	No	245 (54)	243 (53.6)	488 (53.8)
Rabies awareness	Yes	370 (81.5)	400 (88.3)	770 (84.9)
	No	84 (18.5)	53 (11.7)	137 (15.1)
Dog ownership	Yes	156 (34.4)	206 (45.5)	362 (39.9)
	No	298 (65.6)	247 (54.5)	545 (60.1)
	Total	454 (100)	453 (100)	907 (100)

### 3.2 Ecology and demographic characteristics of owned dogs

A total of 6609 dogs (4173 males and 2436 females, with a male-to-female ratio of 1.7:1) were estimated in 42 kebeles. The proportion of dog-owned households (DOHH) was 5.9 %. The average number of dogs per all households was 0.8, while that of DOHH was 1.3. The dog-to-human ratio was 1:48.5 in all households. Both dog-to-household and dog-to-human ratios were higher in rural districts than urban districts (Table 2 and Appendix 2).

**Table 2:** Summary of human and dog populations, dog-to-human and dog-to-household ratios in the Amhara region, Ethiopia (2021 G C.).

Variable	Residence district		
	Rural	Urban	Total
Total human population a^,^^b^.	147158	173655	320813
Total number of HH a^,^^b^	28932	55729	84661
Number of dog-owning HH^b^	3589	1403	4992
Proportion of dog-owning HH^b^	12.4	2.5	5.9
Number of dogs^b^	4819	1790	6609
Average number of dogs per HH^b^	0.17	0.03	0.08
Average number of dogs per dog-owned HH^b^	1.34	1.27	1.32
Dog to human ratio^b^	1: 30.5	1: 97	1:48.5
Dog-to-household ratio^b^	1:6	1:31	1:12.8
Number of male dogs^b^	2950	1223	4173
Number of female dogs^b^	1869	567	2436
Male-to-female dog ratio^b^	1.6:1	2.2:1	1.7:1
Total household^c^	224218	75634	299852
Average number of dogs per district^c^	49042	2390	51432

HH = Household.

a The number of human populations was obtained from the surveyed *kebeles* of the districts, not representing the whole district.

b Data obtained during the census from the selected *kebeles* of all districts.

c Data based on estimated number of households and dog population.

Only 57 % of the owned dogs were confined and 16 % of them vaccinated. The majority of the dog owners (97 %) keep dogs for home guard and livestock herding. The detail of demographic characteristics and ecology of owned dogs' is presented in Table 3.

**Table 3:** Demographic characteristics and ecology of owned dog populations in selected districts of the Amhara region, Ethiopia.

Variable	Category	Number of dogs (%)
Sex	Male	4173 (63)
	Female	2436 (37)
Breed	Local	6478 (98)
	Cross and exotic	131 (2)
Age of dogs	<1 year	988 (15)
	1–3 years	3447 (52)
	>3–6 years	1757 (27)
	>6 years	417 (6)
Source of dog(s)	Gift	4735 (72)
	Homeborn	1309 (20)
	From street	118 (2)
	Bought/purchased	447 (6)
Confinement	Yes	3750 (57)
	No	2859 (43)
Functions/purpose	Only home guarding	5843 (88)
	Only herding	118 (2)
	Both guarding and herding	623 (9)
	Pet/companionship	25 (1)
Vaccination status	Vaccinated	1077 (16)
	Non-vaccinated	5532 (84)
	Total	6609 (100)

### 3.3 Dog management and reproduction

Fifty-four percent of households did not provide separate houses for their dogs. In the remaining households, the dogs are kept in a separate doghouse or tethered in the compound. Forty percent of households provide food for their dogs; of these, 24 % give household leftovers, and the other 24 % provide special prepared food. About 61 % of DOHH had no experience of vaccinating their dogs. The detail of dogs’ management practices is presented in Table 4.

**Table 4:** Management practice of dogs in selected districts of the Amhara region, Ethiopia.

Variables	Category	Frequency	Proportion (%)
Housing practice	Tethering in compound	82	23
	Free in the compound	195	54
	Separate house	85	23
Confinement time	All time	78	22
	Only daytime	121	33
	Not confined	163	45
Feeding practice	Fully owners' hand-fed	199	55
	Partially hand-fed (scavenging)	163	45
Feed source	House leftover	88	24
	Part of family food	144	40
	Special food	88	24
	Mixed	42	12
Feeding frequency per day	Once	12	3
	Twice	112	31
	Three times	176	49
	Four times	13	4
	Not known	49	13
Source of water	Pipe	283	78
	Spring	19	5
	River	46	13
	Others/mixed	14	4
Watering frequency per day	Once	27	8
	Twice	23	6
	Three times	51	14
	Four times	11	3
	Not known	250	69
Vaccination practice	Yes	142	39
	No	220	61
Vaccination by residence	Urban	96	68
	Rural	46	32
Neutering practice	Yes	32	9
	No	330	91
Action on newborn dogs	Give to others	33	40
	Cull	25	30
	Abandon on streets	25	30

**Fig. 2:**
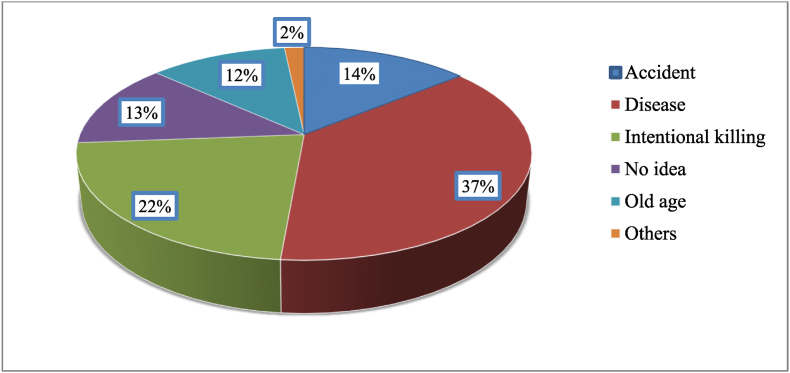
Cause of dogs' deaths according to the respondents. Note: Others include predator, starvation and hunger, food poisoning, etc.

**Fig. 3:**
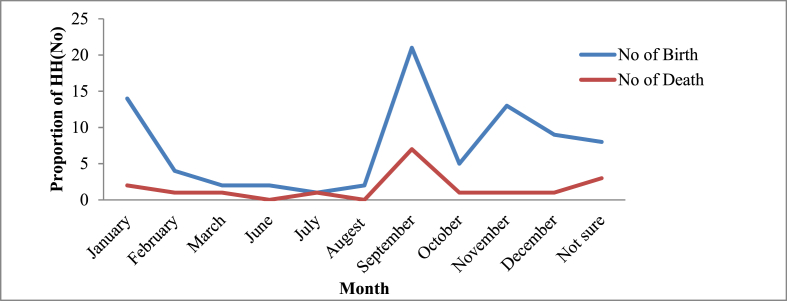
Frequency of births and deaths of dogs across months based on respondents' responses. Note: No = number.

**Fig. 4:**
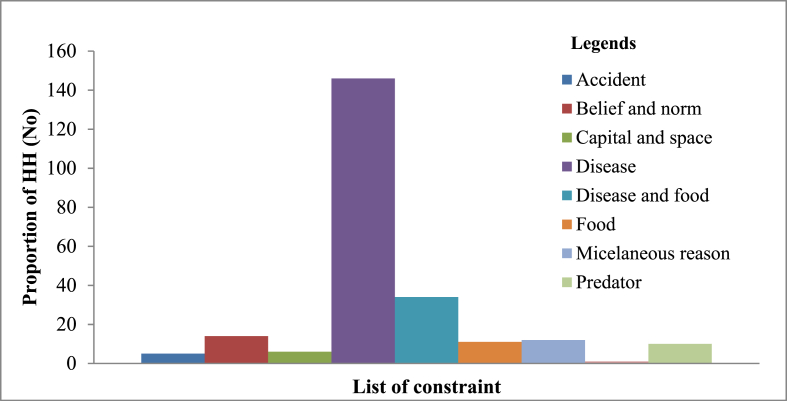
Constraints identified for keeping of dogs based on respondent's response.

Most causes of dog deaths were associated with disease (37 %), followed by intentional killing by the community (22 %) and accidents (14 %) (Fig. 2). The highest numbers of births and deaths were reported to be in September (Fig. 3). From the household members, mothers were responsible (40 %) for managing and caring for dogs. Diseases were mentioned as the main constraint to keep dogs by the respondents (Fig. 4).

A total of 500 puppies were born from 72 pregnant bitches (1–13 puppies per bitch) in the last year. The average fecundity was 6.9 per dog/year. However, only 14.8 % of the puppies were maintained for purpose while the remaining were culled through different means, such as providing to others (40 %) or abandoning on the streets. The average age of male dogs to start mating was 1.4 years, and the mean age of female dogs to give first whelping was 1.5 years. The owner reported that the average life span of dogs was 7.4 years in both sexes. The reproductive and life span data of dogs is presented in Table 5.

**Table 5:** Reproductive parameters and life expectancy of dogs according to the respondent's response.

Variable	Category	Frequency of HH (%)	Mean ± SD	Min	Max
First mating age	<1 year	30 (14.4)	1.4 ± 1.2	0.25 years	3 yrs
	1 year	145 (67)			
	>1–2 years	30 (14.4)			
	>2 years	11 (5.1)			
	Total	216 (100)			
First whelping	<1 year	10 (4.4)	1.5 ± 0.8	0.5 years	6 years
	1 year	133 (58.3)			
	>1–2 years	14 (6)			
	>2 years	71 (31)			
	Total	228(100)			
Life expectancy	5 years	39 (12.9)	7.4 ± 5.7	1 year	27 years
	>5–10 years	142 (46.9)			
	>10 years	122 (40.3)			
	Total	303 (100)			

Non-dog-owned households mentioned their reasons for not keeping dogs: 29 % had no space to keep dogs, 25 % disliked dogs, 24 % were due to fear of disease transmitted by them, 9 % did not feel the necessity of having a dog, 8 % were due to the costs of keeping dogs, and 5 % lacked dog sources. However, 31 % (166/545) of respondents who had no dogs at the time of the survey had interest in keeping a dog in the future.

### 3.4 Factors associated with dog ownership and rabies outbreaks

Residence, zone, age category, sex, household size, religion, occupation, educational status, marital status, income level, year lived in the area, cat ownership, livestock ownership, and rabies awareness showed significant association with dog ownership in univariable analysis (Table 6) and were tested further in multivariable mixed-effect logistic regression models. Zone, religion, livestock ownership, and occupation were significantly associated (P < 0.05) with dog ownership in the multivariable mixed-effect logistic regression models (Table 6).

**Table 6:** Summary of univariable (p < 0.25) and multivariable (p < 0.05) analysis of factors and dog ownership using a mixed-effect logistic regression models including *kebele* and village as random effect variables.

Variable	Category	Total No. of HH surveyed (n = 907)	No. of dog-owned HH (%)	Univariable		Multivariable	
				AOR (95 % CI)	P-value	AOR (95 % CI)	P- value
Residence	Rural	453	206 (45.5)	Ref	–	–	–
	Urban	454	156 (34.4)	0.6 (0.5–0.8)	0.001	–	–
Zone	Central Gondar	161	82 (50.9)	Ref	–		
	North Gondar	154	60 (39)	0.6 (0.4–0.9)	0.33	0.9 (0.4–2.2)	0.86
	North Wollo	152	38 (25)	0.3 (0.2–0.5)	0.000	0.24 (0.1–0.5)	0.001
	South Gondar	151	49 (32.5)	0.5 (0.3–0.7)	0.001	0.4 (0.2–0.9)	0.02
	West Gondar	148	71 (48)	0.9 (0.6–1.4)	0.6	0.8 (0.3–1.7)	0.5
	Wag-Himra	141	62 (44)	0.8 (0.5–1.2)	0.23	0.7 (0.3–1.5)	0.34
Age	Adult (>30–60 years)	147	64 (43.5)	Ref	–	–	–
	Young (<18 years)	48	27 (56.3)	1.9 (1.04–5)	0.04	–	–
	Youth (>18–30 years)	276	95 (34.4)	0.8 (0.6–1.1)	0.11	–	–
	Old (>60 years)	436	176 (40.4)	1.2 (0.8–1.7)	0.5	–	–
Sex	Female	330	118 (35.8)	Ref	–	–	–
	Male	577	244 (42.3)	1.3 (1.0–1.75)	0.047	–	–
Household size	Small (<3 persons)	151	30 (19.9)	Ref	–	–	–
	Medium (3–6 persons)	474	176 (37.1)	2.4 (1.5–3.7)	0.000	–	–
	Large (>6 persons)	282	156 (55.3)	5 (3.1–8)	0.000	–	–
Religions	Muslims and others	55	9 (17.3)	Ref	–	Ref	–
	Orthodox Christian	849	353 (41.6)	4.3 (2.4–9)	<0.001	3.7 (1.6–8.3)	0.001
Occupation	Private	108	31 (28.7)	Ref		Ref	–
	Farmer	417	215 (51.6)	1.5 (0.8–2.8)	0.19	1.3 (0.8–1.8)	0.004
	Government employee	144	40 (27.8)	0.5 (0.3–1.1)	0.087	0.5 (0.13–2.2)	0.39
	Business	154	37 (24)	0.4 (0.2–0.9)	0.024	0.3 (0.07–1.2)	0.08
	Others	84	39 (46.4)	1.2 (0.6–2.5)	0.57	0.6 (0.12–2.7)	0.48
Educational level	Illiterate	300	132 (44)	Ref	–	–	–
	Primary school	287	118 (41)	0.9 (0.6–1.2)	0.48	–	–
	Secondary school	178	74 (41.6)	0.9 (0.6–1.3)	0.605	–	–
	College and above	142	38 (26.8)	0.5 (0.3–0.7)	0.001	–	–
Marital status	Divorced∗	58	14 (24.1)	Ref	–	–	–
	Married	626	264 (42.2)	2.3 (1.2–4.3)	0.009	–	–
	Single	189	75 (39.7)	2.1 (1.1–4.03)	0.033	–	–
	Windowed	34	9 (26.5)	1.1 (0.4–3)	0.803	–	–
Income level	Not known	470	196 (41.7)	Ref	–	–	–
	<1500 ETB	142	45 (31.7)	0.8 (0.6–1.1)	0.15	–	–
	1500-4000 ETB	246	103 (41.9)	0.9 (0.7–1.4)	0.9	–	–
	>4000 ETB	49	18 (36.7)	0.5 (0.3–0.9)	0.09	–	–
Years in the area lived	20–30 years	192	84 (43.8)	Ref	–	–	–
	5 years	146	42 (28.8)	0.5 (0.3–0.8)	0.007	–	–
	5–10 years	116	43 (37.1)	0.8 (0.5–1.2)	0.24	–	–
	10–20 years	206	96 (46.6)	1.1 (0.8–1.7)	0.6	–	–
	>30 years	247	96 (38.9)	0.8 (0.6–1.2)	0.3	–	–
Cat owned	No	488	158 (32.4)	Ref	–	–	–
	Yes	419	204 (48.7)	2 (1.5–2.6)	0.000	–	–
Livestock owned	Yes	495	280 (56.6)	5.2 (3.9–7.1)	0.000	5.9 (3.9–8.2)	0.001
	No	412	82 (19.9)	1.0	–	Ref	–
Rabies awareness	No	137	43 (31.4)	Ref	–	–	–
	Yes	770	319 (41.4)	1.5 (1–2.3)	0.028	–	–

**Note:** Ref **=** Reference category; HH = Household; COR = Crude Odd Ratio; AOR = Adjusted Odd Ratio; CI = Confidence Interval.

Of the total 907 households surveyed, 15.3 % (95 % CI: 13.04%–17.8 %) (139/907) households had a history of rabies outbreak experience either in humans or in their livestock within the last 5 years. Of these, 15 % (81/545, 95 % CI: 12.3%–20.5 %) were non-DOHH and 16 % (58/362, 95 % CI: 14.3%–22.5 %) were DOHHs. Across residence, 18.3 % were in rural and 12.3 % were urban households.

Residence, zone, educational status, age of respondents, occupation, income level, and livestock ownership showed significant association to having observed the occurrence of rabies at a p-value of 0.25 in univariable mixed-effect logistic regression models (Table 7) and are tested further in multivariable mixed-effect logistic regression models. Residence, age, and zone were associated (p < 0.05) with having observed the occurrence of rabies in the multivariable mixed-effect logistic regression models (Table 7).

**Table 7:** summary of univariable (p < 0.25) and multivariable (p < 0.05) analysis of putative risk factors for rabies occurrence using mixed-effect logistic regression model including *kebele* and village as random effect variables.

Variable	Category	No. of HH surveyed (n = 907)	HH with rabies case history (%)	Univariable		Multivariable	
				COR (95 % CI)	P-value	AOR (95 % CI)	P-value
Residence	Rural	453	83 (18.3)	Ref	–	Ref	–
	Urban	454	56 (12.3)	0.6 (0.4–0.9)	0.013	0.1(0.02–0.5)	0.004
Zone	Central Gondar	161	36 (22.4)	Ref	–	Ref	–
	North Gondar	154	19 (12.3)	0.5 (0.3–0.9)	0.021	0.5 (0.2–1.1)	0.07
	North Wollo	152	24 (15.8)	0.7 (0.4–1.2)	0.142	6.4 (1.1–36)	0.036
	South Gondar	151	18 (11.9)	0.5 (0.3–0.9)	0.016	0.5 (0.2–1.1)	0.108
	West Gondar	148	23 (15.5)	0.6 (0.4–1.1)	0.129	0.3 (0.1–0.6)	0.003
	Wag-Himira	141	19 (13.5)	0.5 (0.3–1)	0.048	0.3 (0.1–0.8)	0.016
Education level	Illiterate	300	42 (14)	Ref	–	–	–
	Primary school	287	49 (17.1)	1.8 (0.7–5)	0.23	–	–
	Secondary school	178	32 (18)	1.3 (0.4–4.2)	0.67	–	–
	College and above	142	16 (11.3)	3.3 (1.1–10)	0.04	–	–
Age	>30–60 years	436	79 (18.1)	Ref	–	Ref	–
	<18 years	48	4 (8.3)	0.4 (0.1–1.2)	0.09	0.4 (0.13–1.4)	0.004
	18–30 years	276	31 (11.2)	0.6 (0.4–0.9)	0.014	0.5 (0.3–0.8)	0.157
	>60 years	147	25 (17)	0.9 (0.6–1.5)	0.76	1.1 (0.7–1.9)	0.67
Occupation	Private	108	18 (16.7)	Ref	–	–	–
	Farmer	417	79 (19)	1.1 (0.5–2.5)	0.79	–	–
	Governmental	144	17 (11.8)	0.6 (0.3–1.6)	0.33	–	–
	Business	154	19 (12.3)	0.7 (0.3–1.6)	0.38	–	–
	Others	84	6 (7.1)	0.4 (0.12–1.1)	0.08	–	–
Income level	Not known	470	61 (13)	Ref	–	–	–
	<1500 ETB	142	29 (20.4)	1.5 (0.9–2.2)	0.06	–	–
	1500-4000 ETB	246	43 (17.5)	1.5 (0.8–2.4)	0.15	–	–
	>4000 ETB	49	6 (12.2)	1.5 (0.9–2.2)	0.08	–	–
Livestock owned	No	412	46 (11.2)	Ref	–	–	–
	Yes	495	93 (18.8)	1.8 (1.3–2.7)	0.002	–	–

**Note**: Ref = reference category; HH = household; COR = crude odds ratio; AOR = adjusted odds ratio, CI = confidence interval.

## 4 Discussion

In this study, the estimation of owned dogs and the households who owned dogs was larger in rural than in urban districts. This is in line with other previous reports [26,27]. This can be explained by the fact that dogs are more useful in rural communities than urban communities. However, this proportion of dogs could be changed if the stray dogs were included. Of note, the number of stray dogs is larger in urban areas than in rural areas [27,28].

The mean number of dogs per DOHH (1.3) in the current study was within the range of the mean number of dogs per DOHH reported previously: 1.1 reported from the West Shewa zone [26] and 1.50 and 2.05 reported, respectively, in urban and pastoralist areas from the Awash Basin, eastern Ethiopia, Tschopp et al. [25]. The percentage of dog-owned households (5.9 %) is lower than the percentage of dog-owned households reported from various countries; for instance, 88.9 % in Madagascar [3], 82 % in Harare, Zimbabwe [29], 63 % in Machakos district in Kenya [30], 11 % from Zambia [31], and 7.1 %–15.1 % from Tanzania [27]. Similarly, higher dog ownership percentages than the current study were also reported out of Africa: 24.2 % from Japan [32], 65.2 % from Mexico [33], and 42.7%–56.4 % from Mexico City [34], 54 % from California [35], and 73 % from Merida, Yucatan [36]. The variation among the reports could be due to the differences in socio-cultural, economic, and attitudes towards dog ownership [[25], [26], [27]].

A higher proportion (76 %) of DOHH keep one dog, which is comparable to the work of Gebremedhin et al. [26], who reported that 74.8 % of DOHH kept one dog. The average overall dog-to-human ratio (1:48.5) calculated in the current study was lower than the dog-to-human ratio reported from Ethiopia previously: 1:4.7 reported by Tschopp et al. [25] and 1:6 reported by Gebremedhin et al. [26] A higher dog-to-human ratio has been reported from other African countries, including Tanzania 1:14.4 to 1:27.2 [27], Maboloko, Botswana 1:11 [37], Kikambuani, Kenya 1:15 [36], Zimbabwe 1:16 [38], N'Djaména, Chad 1:21.5 [39], and Zambia 1:6.7 [31]. The decline in the dog-to-human ratio can be explained by the decline in keeping dogs associated with fear of rabies. We have shown that a larger proportion of respondents (84.9 %) are aware of the presence of rabies. Moreover, in addition to the shortage of space and disliking dogs, fear of disease transmitted by dogs was a reason for not keeping dogs.

The male dogs to female dogs ratio is in line with the work of Gebremedhin et al. [26] and Tschopp et al. [25] from Ethiopia, who reported male-to-female ratios of 3:1 from West Shewa, Ethiopia, and 1.6:1 and 1.5:1 from the Methara and Oromia districts of Eastern Ethiopia, respectively. The finding of male dogs' dominance in the current study supports reports from other countries: 2.2:1 in Kwara State, Nigeria [40], 1.3–5.3:1 in Chile [41], 1.6:1 in Madagascar [3], and 2:1 in Thailand [42]. This could be explained by the fact that many people did not like female dogs for having unwanted litters and being the cause of the gathering of many male dogs for mating. The belief that male dogs are better guardians and hunters than bitches and the higher mortality in female dogs [26,[43], [44], [45]] can also contribute to the dominance of male dogs. However, a higher bitch-to-male dog ratio has been reported from Afar in Ethiopia [25] and outside Ethiopia [21,46].

The average litter size (6.9) recorded in the current study was larger than the average number of puppies reported from other countries (5.5 and 4.7) from Iringa, Tanzania's urban and rural districts, respectively [28,47], and 4.6 and 4.8 pups in Zimbabwe [38,47]. The finding of larger litter is a concern because it can be a cause for increasing stray dogs. The mean ages of first mating of both male and female dogs were slightly greater than the mean ages of first mating in dogs reported by Gsell et al. [28], who reported that the first mating age of female dogs was 10 months. This was not surprising because sexual maturity in dogs reaches around six months to one year for both males and females; even this can be delayed until two years of age for some larger breeds depending on genetic and environmental factors [48].

The 7.4-year average life expectancy of dogs reported by owners in the current study is lower than the life expectancy of 12.3 years reported by Gebremedhin et al. [26] and the 10–13 years [49] and 8.3 years [50] life expectancy reported from other countries. Nonetheless, life expectancy less than the life expectancy estimated in the current study was reported: 1.9 years in Serengeti [51], 2.76 years in Tanzania [28], 2.8 years in Machakos [30], 4.5 years in North America [52], 5.5 years in Nigeria [21], and 3.7 years in Zimbabwe [38]. All these authors explained that the average life expectancy of an animal is determined by genetic makeup, metabolic rate, body size, and disease condition.

Nine percent of DOHH castrated or spayed their dogs, unlike in the developed world where neutering of female dogs was considered a major means of dog population control [53]. Respondents suggested that educating society not to release dogs and free-roaming and killing stray dogs is a means of controlling dog population. From the current finding, it is possible to understand that controlling reproduction of dogs was not a widespread practice despite it being an essential part of dog management. Dog overpopulation with poor vaccination coverage and scavenging poses risks to the community in causing physical risks, spreading infections like rabies to people and their livestock [54].

Forty-five percent of DOHH allowed dogs to move free. This finding indicates that a little bit better dog management is compared to other African countries reported; 62 %–79 % of owned dogs are allowed to roam free [3,55,56]. This might be related to differences in socio-economic status and attitudes between societies regarding dog management and differences in awareness of dogs’ welfare [26].

Anti-rabies vaccination coverage (39 %) estimated in the current study was higher than the anti-rabies vaccination coverage in urban areas in the country (20 %), but it still falls below 70–80 %, the World Health Organization recommendation of vaccination of dogs [57]. It was also lower than the 69.6 %, 64.1 %, and 49.5 % reported in Niger state [55], Lagos [46], and Abuja [58], respectively. Anti-rabies vaccination coverage lower than the finding in the current study was also reported: 26.4 % and 21 % were reported in Bauchi [59] and Nasarawa state [21], respectively. This could be associated with a lack of awareness about the anti-rabies vaccine and its accessibility and socioeconomic status among different countries and societies. It was reported that dog owners who were willing to vaccinate their dog at their own cost were very few [19]. This can be supported by the finding in the current study that higher anti-rabies vaccination coverage of dogs in the urban communities than in the rural communities. Moreover, the absence of an official rabies control program contributes to the low anti-rabies vaccination coverage below the World Health Organization recommendation of vaccination of dogs.

The 15.3 % rabies occurrence estimated in the current study is higher than the report from previous studies in Ethiopia and from other countries [9,[60], [61], [62], [63], [64], [65], [66]]. The higher rabies outbreak may indicate that the existing control and prevention methods are not adequate and/or less functional. However, as the number of rabies outbreaks was based on the respondents’ experiences and know-how level of rabies cases, the actual number of rabies occurrences might be either over- or underestimated. Childs et al. [67] outlined that the accurate estimates of rabies outbreaks from questionnaire data are difficult to obtain in developing countries because of poor surveillance systems and inadequate regional laboratories in these countries.

The odds of owning a dog were higher in Orthodox Christians than in Muslims and other religious groups. This is in line with the findings of Oboegbulem and Nwakonobi [68] and Mauti [69]. This is not surprising because dogs are considered impure, and dog ownership is proscribed in Muslim religious belief [70]. In addition, the numbers of Muslim and other participants were few in this study, which might change the ownership proportion. The odds of dog ownership were higher in individuals who owned other domestic animals too. This finding is in line with the findings in the previous reports [26,71]. This could be explained in relation to the purpose of keeping dogs for guarding livestock. Farmers have higher odds of dog ownership than self-employed respondents. This could be related to the nature of the occupation of farmers, as their livelihood is based on keeping livestock both for production and planting crops, and most farmers reside in rural areas.

The higher odds of rabies outbreak in rural residents than urban residents were in line with the findings of Gebru et al. [66] and Yizengaw et al. [72]. This can be explained in relation to the larger number of dogs and low anti-rabies vaccination coverage we found in the current study and low awareness of anti-rabies vaccination in rural residents compared to residents in urban areas. The odds of rabies outbreak were higher in the North Wollo zone but lower in the North Gondar, West Gondar, and Wag-Hemra zones compared to the odds of rabies outbreak in the Central Gondar zone. This can be explained in relation to differences in the population of dogs, differences in public awareness about rabies, or differences in anti-rabies dog vaccination coverage [65,66]. The odds of having observed rabies occurrence were lower in humans of age groups less than 18 years than in humans of age groups >30–60 years; this contradicts with previous reports [16,65,66]. This could be due to the fact that respondents less than 18 years of age have less time and probability to observe rabies occurrences in humans or animals as compared to older individuals in this study, but it can partly be due to socio-cultural differences among various communities. For instance, in some communities, adults may be at high risk of rabies exposure due to the fact that they usually conduct their outdoor activities in distant places away from their home. On some other communities, children might be more commonly exposed to rabies while they are playing with dogs or because they may not be well attended [66].

## 5 Conclusions

The study provides knowledge of owned dog demography and ecology. The total number of dogs, the male-to-female ratio of dogs, the dog-to-human ratio, the average number of dogs per household, and the proportion of dog-owning households were low compared to previous reports, although there was variation between districts and zones. This might make dog-based control of rabies, such as vaccination, feasible. Most dogs were not confined and vaccinated, which indicates the need for more awareness on responsible dog ownership. Dog ownership had no different impact on the rabies outbreak. Although further study is needed at some points, many factors, such as zone, residence, and religion, are associated with dog ownership and rabies outbreaks, which can be helpful in tailoring rabies control in the Amhara region. Creating community awareness about rabies is important to promote dog vaccination coverage. Generally, the study provides useful information about the demography and ecology of owned dogs in relation to rabies for making proper and effective rabies control strategies and is important to design the spatial distribution of rabies vaccination in dogs. Moreover, detailed longitudinal studies about rabies outbreak occurrence, dog demography and ecology, including stray dogs, different species of animals, and communities such as pastoralists, are suggested.

## CRediT authorship contribution statement

**Liuel Yizengaw Adnie:** Writing – review & editing, Writing – original draft, Validation, Investigation, Formal analysis, Data curation, Conceptualization. **Wudu Temesgen Jemberu:** Writing – review & editing, Writing – original draft, Validation, Supervision, Project administration, Methodology, Funding acquisition, Data curation, Conceptualization. **Adane Bahiru Woreta:** Writing – original draft, Investigation, Data curation. **Adugna Berju:** Visualization, Investigation, Data curation. **Araya Mengistu:** Writing – original draft, Validation, Methodology, Formal analysis, Conceptualization. **Zeleke Tesema Wondie:** Writing – original draft, Validation, Methodology, Investigation, Formal analysis, Data curation. **Wassie Molla:** Writing – review & editing, Writing – original draft, Validation, Supervision, Project administration, Methodology, Investigation, Funding acquisition, Formal analysis, Data curation, Conceptualization. **Sefinew Alemu Mekonnen:** Writing – review & editing, Writing – original draft, Supervision, Resources, Project administration, Methodology, Investigation, Funding acquisition, Formal analysis, Data curation.

## Ethics approval and consent to participant

The study was approved by the University of Gondar Research and Technology Transfer Ethical Committee (VP/RRTT 05/1037/2022). The participants have given verbal informed consent for their participation in the study (VP/RTT 05/1037/2022). Verbal informed consent was approved by the University of Gondar Research and Technology Transfer Ethical Committee. For illiterate participants, the consent was provided through their legally authorized representatives based on the surrounding community rule and regulation (*kebele* and village leaders), who have legal responsibility and right in such kind of issue as usual. Consent form for participation and ethical consideration found in supplementary material as appendix 4. All the methods were carried out in accordance with relevant guidelines and regulations. Consent form for participation and ethical consideration found in supplementary material as appendix 4.

## Data availability statement

Data will be made available on request.

## Funding statement

This study was supported by 10.13039/501100007861University of Gondar and Amhara Region Agricultural Research Institute (CVMAS/13/313/2013).

## Declaration of competing interest

The authors declare the following financial interests/personal relationships which may be considered as potential competing interests: Reports a relationship with that includes: Has patent pending to. If there are other authors, they declare that they have no known competing financial interests or personal relationships that could have appeared to influence the work reported in this paper.

## Footnotes

Appendix A Supplementary data to this article can be found online at https://doi.org/10.1016/j.heliyon.2024.e41582.
