# Supplementary material for: Dog demography and ecology with reference to rabies in the Amhara region, Ethiopia
Source: Heliyon. 2024 Dec 31;11(1):e41582. doi: 10.1016/j.heliyon.2024.e41582 (PMC11755056; doi:10.1016/j.heliyon.2024.e41582)
Supplement: Multimedia component 1 [file mmc1.docx]

# Supplementary Materials

Appendix: 1. Questionnaire from sampled dog ecology survey in selected household.

**Enumerator Name ____________________Signature __________Date ___________**

**General information of the Study area**

1. Administrative zone __________________
2. District ____________
3. Kebele___________
4. Village ___________
5. Geo-reference of the village: longitude ______ latitude ______altitude______
6. Agro-ecology: high land _________ mid-land___________ low land________

**Part I: Respondent socio-demography information**

- 1. Respondent: Name _______________________________Sex _____Age_____yrs.
  2. Are you the head of household? A/ Yes B/ No
  3. If Q4 answer is no, what is the relation to head of the household?

A/ Children B/ Sister and Brother C/ Other relatives

- 1. Educational status:

A/ Illiterate B/ Primary school (1-8) C/ Secondary school (9-12) D/ College and Above

- 1. Religion: ( Tick √ sign): Orthodox ______, Muslims _____, Protestant______, Others_______
  2. Income status per month: ______________ Ethiopian birr
  3. Marital status: ( tick √ sign): Single _____ Married _________Divorced____ Widowed____
  4. Total number of people living in household: _______________.
  5. Characterization of individual in household

| Head of household (√) | Age | Sex and Age of children | | | |
| --- | --- | --- | --- | --- | --- |
| Male (husband) _____ |  | **Female** | **No** | **Male** | **No** |
|  |  | Female < 18 yrs |  | Male < 18 yrs |  |
| Female (wife) _______ |  | Female < 19- 30 yrs. |  | Male < 19- 30 yrs. |  |
|  |  | Female < 31-40 yrs |  | Male < 31-40 yrs |  |
|  |  | Female > 40 yrs |  | Male > 40 yrs |  |

- 1. Residence (Geographical background): A/ Rural B/ Urban
  2. How long you lived in the area? __________yrs.
  3. Occupation: ( tick √ sign)

Farmer ____ Business man____ Governmental employ____ Others (Specify) _________

- 1. Have you livestock? A/ Yes B/ No
  2. If Q15 answer is yes, list species of livestock you keep currently

1, _______ 2, ________ 3, ________, 4, _______ 5, ______ 6, ________ 7, _______

- 1. Do you know rabies? A/ Yes B/ No
  2. Have you experienced the rabies occurrence or case in the last 5 years in your family or animals? A/ Yes B/ No
  3. If Q 18 answer is yes, in which month the most case of rabies occurred? Rank it.

| Month | Sep. | Oct. | Nov | Des | Jan | Feb | Mar | Apr | May | Jun | Jul | Aug |
| --- | --- | --- | --- | --- | --- | --- | --- | --- | --- | --- | --- | --- |
| **Rank** |  |  |  |  |  |  |  |  |  |  |  |  |

- 1. If Q 18 yes, what are the causes/source of the rabies? ___________, ________, ________
  2. Do you think the source of rabies is only dog? A/ Yes B/ No
  3. Do you have owned cat? A/ Yes B/ No
  4. Do you have owned dogs? A/ Yes B/ No
  5. If Q 23 answers is no, what is the reason?

A, Do not like B, Have no space C, Require much time and money

D, source of disease like rabies E/ Others

- 1. Have you planned having own dogs in the next 12 month? A/ Yes B/ No

**Part II: Dog demographics and population dynamics of the dog owned respondents**

What/ where is the source of your dogs? ( **tick √ sign**)

A/ Gift/ received B/ Bought C/Neighbor D/ Left on Street E/ Others

The age of dog when you acquired at the first time _____ month/ year.

How long you owned the dog ?________years

Sex of your dog /s: A/ Male B/ Female C/ Both

Total number of dogs owned by household: ______________

| **No** | Sex | Age | Breed | Confinement | | Altered | | Functions/ Purpose of owning | Vaccination status | |
| --- | --- | --- | --- | --- | --- | --- | --- | --- | --- | --- |
|  |  |  |  | Yes | No | Yes | No |  | Yes | No |
|  |  |  |  |  |  |  |  |  |  |  |
|  |  |  |  |  |  |  |  |  |  |  |
| Total |  |  |  |  |  |  |  |  |  |  |

1. Number of puppy/ dogs born in the last **one** year

| No of puppy born | Sex | | Breed | | | **Total** |
| --- | --- | --- | --- | --- | --- | --- |
|  | M | F | Local | Exotic | Cross |  |
| **No** |  |  |  |  |  |  |

1. In which month the highest birth rate (whelping) occurred? **Rank it**?

| Month | Sep. | Oct. | Nov. | Des. | Jan. | Feb. | Mar. | Apr. | May | Jun. | Jul. | Aug. |
| --- | --- | --- | --- | --- | --- | --- | --- | --- | --- | --- | --- | --- |
| Rank |  |  |  |  |  |  |  |  |  |  |  |  |

1. How many of the puppies are raised for home and what purpose

| **Category** | **Sex** | | **Breed** | | | **Purpose/ Reasons** |
| --- | --- | --- | --- | --- | --- | --- |
|  | M | F | Local | Exotic | Cross |  |
| **No** |  |  |  |  |  |  |
| **Total** |  | |  | | |  |

1. What happens to the remaining puppies (the fate of puppy)?

A/ Culled B/ Give to others C/ Sold D/ Leave on street E/ Others

1. Number of dog died in the last **one year**

| Category | **Sex** | | **Age** | | | **Breed** | | | **Total** |
| --- | --- | --- | --- | --- | --- | --- | --- | --- | --- |
|  | M | F | < 6mth | 6-12 mth | > 1 yrs | Local | Exotic | Cross |  |
| **No** |  |  |  |  |  |  |  |  |  |
| **Total** |  | |  | | |  | | |  |
| **Cause (**if known) |  | |  | | |  | | |  |

1. In which month the highest death rate occurred? **Rank it**?

| Month | Sep. | Oct. | Nov | Des | Jan | Feb | Mar | Apr | May | Jun | Jul | Aug |
| --- | --- | --- | --- | --- | --- | --- | --- | --- | --- | --- | --- | --- |
| Rank |  |  |  |  |  |  |  |  |  |  |  |  |

1. What is the main cause of dog death?

A/ Natural death (old age) B/ Intentionally killed C/ Death due to disease

D/ Accident E/ Dog control by municipality F/ others (mentions)

1. How many years do your dog’s live (from past experience).

Female ______years Male _______years

1. The mean/ average first littering age of bitch __________yrs.
2. The mean/ average number of live pups per littering _____ (**if so)** female ____male ___))
3. The time interval taken between two consecutive birth (littering interval).____month/yrs.
4. The average age of male dog starting to mate/ breed ________yrs.
5. How many times the bitch gives birth (littering) in lifetime _________yrs.
6. The average reproductive age of bitch giving birth __________yrs.
7. No of bitch currently pregnant __________.
8. No of bitch pregnant during the last 1 year ____________.
9. The average reproductive age of male dog giving service ___________yrs.
10. What are the main constraints in rearing dog? **Rank** it.

| **Constraints** | **Rank** | **Constraints** | **Rank** |
| --- | --- | --- | --- |
| Disease |  | Norm/ beliefs( religions) |  |
| Food / feed shortage |  | Predators (hyena) |  |
| Capital |  | Others |  |
| Breed/ genotype |  |  |  |

1. What is the trend of dog population in the last 5-10 years**(encircle)**

A/ Increase B/ Decrease C/ Constant D/ Not Sure

1. What are the reasons for population change/ dynamics **and rank**

| **Reason for increasing** | **Rank** | **Reason for decreasing** | **Rank** |
| --- | --- | --- | --- |
| Due to their function |  | Due to their function loss |  |
| Used as source of income |  | Source of disease |  |
| Entertainment |  | Feed requirement( shortage) |  |
| Others(specify |  | Extra space requirement |  |
|  |  | Others (specify) |  |

**Part III: The management practice of owned dog**

1. Who takes care of the dogs?

A/ Father B/ Mother C/ Children D/ Others relatives

1. What is the confinement status of your dog?

A/ All-time B/ only during day C/ only during night D/ not confined

1. Type of housing: A/ Tethering in the compound B/ Separate Dog House

C/ No house (corridor) D/other (mention)

1. Is there any contact between your dog and others? A/ Yes B/ No
2. How fed your dogs?

A/ Total hand fed B/ Partial hand fed C/ Total scavenging

1. If scavenging where and what do dogs scavenge?

A/ around in the Compound B/ On Street C/ other (mention)

1. If total hand fed, the food source is?

A/ Household left over B/ Special prepared feed/food C/ Part of family food D/ Abattoir by product E/ Others (specify) __________

1. If hand fed, the fed frequency per day

A/ Once B/ Twice C/ Three Time D/ Four Times E/ Not Known

1. What was the source of water for your dogs?

A/ Pipe Water B, River C/ Spring D/ Others (mentions)

1. The frequency of watering for your dogs per day?

A/ Once B/ Twice C/ Three times D/ Four Times E/ Not known

1. The distance of water source on your dogs?

A/ Near at home B/1-5 Km from home C/ 6-10 Km from home

1. Do you practice dog neutering / spaying? A/ Yes B/ No
2. If Q 12 answer is yes which sex? A/ Male B/Female C/ Both
3. Where these practices take place?

A/ At home B/ Private Vet clinic C/ Gov`t Vet clinic D/ others (specify) ______.

1. If Q 12 answer is no what is the reason:

A/ Not knowing B, Need cost C, Risk of surgery injury D, Want to breed

E/ others (specify) _________

1. Is there other dogs bitten by your dog? A/ yes B/ no C/ Not know
2. Is there any person bitten by your dog? A/ yes B/ no C/ Not know
3. Is there vaccination practice for your dogs?

A/ Yes B/ No C/ Not Sure

1. If Q 18 answers is no what the reason was? 1, ____________ 2, _______________
2. If Q 18 answers is yes, the frequency ___________.
3. Where vaccination is takes place?

A/ Private Vet Clinic B/ Gov`t Vet Clinic C/ Home (Traditional)

D/ Both B & C E/ All F/ others (specify) _________________.

1. What type of evidence you used for vaccination history?

A/ Record Book B/ Certificate of Vaccination C/ Oral Declarations D/ Others (mentions) __________, ____________, ___________

1. Have you practice other health care management for your dogs like deworming and other disease prevention and control measure? A/ Yes B/ No
2. If Q 23 answers is yes, did your dog was dewormed in the last period/ 1 year?

A/ YES B/ No

1. If Q 23 answers is **no** what were the reasons? 1, _______ 2, _______, 3,____________
2. Is there any wild Canid in your area? A/ Yes B/ No
3. If Q 26 is yes, please mention them : 1, ___________, 2,__________, 3, _____________
4. If Q 26 is yes, is your dog has been contacted with them? A/ Yes B/ No
5. Have you encountered rabies case in your dog in the last 5 years?

A/ Yes B/ No

1. If Q 30 answer is yes, in which month the most case of rabies occurred? Rank it.

| 1. Moth | Sep. | Oct. | Nov | Des | Jan | Feb | Mar | Apr | May | Jun | Jul | Aug |
| --- | --- | --- | --- | --- | --- | --- | --- | --- | --- | --- | --- | --- |
| Rank |  |  |  |  |  |  |  |  |  |  |  |  |

1. What type of measure you take when dog show rabid?

A/ Culled immediately B/ Leave till death C/ Keep isolated D/ Taking to vet clinic E/ Others (specify)

Appendix 2: Description dog population in selected districts of Amhara region, Ethiopia (from census)

| Variables | | | | | | | | | | | | | |
| --- | --- | --- | --- | --- | --- | --- | --- | --- | --- | --- | --- | --- | --- |
| Zones | C/ Gondar | | N/ Gondar | | N/ Wollo | | S/ Gondar | | W/ Gondar | | Wag -himra | |  |
| Residence/ community type | Rural Sanja | Urban/ koladiba | Rural Janamora | Urban Debarik | Rural Raya kobo | Urban Woldia | Rural Fogera | Urban D/tabor | Rural Metema | Urban G/wuha | Rural Ziquala | Urban Sekota | Total |
| Total human population*. | 12236 | 10783 | 31708 | 32000 | 27519 | 37115 | 39326 | 47868 | 18459 | 21543 | 17910 | 24346 | 320813 |
| Total number of owned dogs* | 854 | 246 | 652 | 513 | 334 | 197 | 1431 | 342 | 852 | 315 | 696 | 177 | 6609 |
| Total number of HH * | 2288 | 2806 | 7927 | 10667 | 6115 | 14242 | 6261 | 15598 | 3356 | 8525 | 2985 | 3891 | 84661 |
| Number of dog owning HH* | 557 | 151 | 565 | 413 | 278 | 144 | 1144 | 283 | 499 | 262 | 546 | 150 | 4992 |
| Proportion of dog owning HH* | 24.3 | 5.3 | 7.2 | 3.8 | 4.5 | 1 | 18.3 | 1.8 | 14.9 | 3.1 | 18.3 | 3.8 | 5.9 |
| Human to dog ratio* | 14.3:1 | 43.8:1 | 48:1 | 62.4:1 | 82.4:1 | 188:1 | 27.5:1 | 140:1 | 21.6:1 | 68.4:1 | 25.7:1 | 132:1 | 48.5:1 |
| Household to dog ratio* | 2.7:1 | 11:1 | 12.2:1 | 20.8:1 | 18:1 | 72.3:1 | 4.4:1 | 45.6:1 | 3.9:1 | 27:1 | 3.9:1 | 22:1 | 13:1 |
| Average number of dogs per dog owning HH | 1.5 | 1.6 | 1.2 | 1.3 | 1.2 | 1.4 | 1.3 | 1.2 | 1.7 | 1.2 | 1.3 | 1.2 | 1.32 |
| Number of male dogs* | 445 | 165 | 462 | 345 | 241 | 142 | 915 | 223 | 506 | 205 | 381 | 143 | 4173 |
| Number of female dogs* | 409 | 81 | 190 | 168 | 93 | 55 | 516 | 119 | 346 | 110 | 315 | 34 | 2436 |
| Male to female dogs ratio* | 1:1 | 2:1 | 2:1 | 2:1 | 3:1 | 3:1 | 2:1 | 2:1 | 1.5:1 | 2:1 | 1.2:1 | 4:1 | 1.7:1 |
| Average number of dogs per HH | 0.37 | 0.09 | 0.08 | 0.05 | 0.06 | 0.02 | 0.3 | 0.02 | 0.3 | 0.04 | 0.3 | 0.05 | 0.08 |
| Total household ** | 40680 | 3083 | 36361 | 11940 | 54466 | 21472 | 52905 | 25958 | 29378 | 9123 | 10428 | 4058 | 299852 |
| Average number of dog per district** | 15052 | 278 | 2909 | 597 | 3268 | 429 | 15872 | 519 | 8813 | 364 | 3128 | 203 | 51432 |
| Density per km^2^ | 2.1 | - | 1.7 | - | 1 | - | 14.8 |  | 2.3 | - | 1 | - | - |

HH= Household

^1 1^The number of human population was that of the surveyed *kebeles* of the districts not representing the whole district.

* = Data obtained during census from the selected *kebeles* of all districts

**= Data based on estimated number of households and dog population; - = It indicated that have no area square kilometer information and dog density.

Appendix 3: enumeration of dog population in selected districts of Amhara region, Ethiopia (from census) data sheet

| Owner name | Id No. | Sex | Age | Breed | Source of dog | Purpose/ Function of dog | Confined status | Owner Income per month | Owner Residence | Owner Occupation | Owner educational level | Dog vaccination status |
| --- | --- | --- | --- | --- | --- | --- | --- | --- | --- | --- | --- | --- |
|  | 1 |  |  |  |  |  |  |  |  |  |  |  |
|  | 2 |  |  |  |  |  |  |  |  |  |  |  |
|  | 3 |  |  |  |  |  |  |  |  |  |  |  |
|  | 4 |  |  |  |  |  |  |  |  |  |  |  |
|  | 5 |  |  |  |  |  |  |  |  |  |  |  |
|  | 6 |  |  |  |  |  |  |  |  |  |  |  |
|  | 7 |  |  |  |  |  |  |  |  |  |  |  |
|  | 8 |  |  |  |  |  |  |  |  |  |  |  |
|  | 9 |  |  |  |  |  |  |  |  |  |  |  |
|  | 10 |  |  |  |  |  |  |  |  |  |  |  |
|  | 11 |  |  |  |  |  |  |  |  |  |  |  |
|  | 12 |  |  |  |  |  |  |  |  |  |  |  |
|  | 13 |  |  |  |  |  |  |  |  |  |  |  |
|  | 14 |  |  |  |  |  |  |  |  |  |  |  |
|  | 15 |  |  |  |  |  |  |  |  |  |  |  |
|  | 16 |  |  |  |  |  |  |  |  |  |  |  |
|  | 17 |  |  |  |  |  |  |  |  |  |  |  |
|  | 18 |  |  |  |  |  |  |  |  |  |  |  |

**District: ____________________________, Kebele_________________________________**

**Enumerator Name: _______________________________________Signature ___________ Date: ____ /______ / _______**

## ****Appendix 4:**** Consent form

Questionnaire for dog ecology and demography with reference to Rabies among residents of Amhara Region, Ethiopia*.*

I am ………... From University of Gondar who is doing research on dog ecology and demography with reference to Rabies. Your contribution for achieving the objective of the research is highly valuable, and incase if you don’t want to participate in the interview process you are not obligated to finalize the interview.

I have read the above information sheet or it has been read to me. The study has been explained to me in detail, and I have had a chance to ask questions and received satisfactory answers. I understand that if I have any other questions later about the research or about my rights as a research subject, I can call the Ethical Review Committee. I also understand that I will receive a copy of this Consent Form if I choose to participate.

Please select one:

Do you agree to participate in this survey (note that you can withdraw from the survey at any time)?

 _____ I agree to participate in this project.

 _____ I do not wish to participate in this project.

Name of participant: _________________________

Date: _________________

Signature of participant: _____________________________

(Or thumbprint if the individual cannot write)

WITNESS (Only necessary if the individual cannot read/write):

Name of Witness: ____________________________________

Signature of Witness: _________________________________

Date: _______________

Thank you in advance for your cooperation!!

**Enumerator Name ____________________Signature __________Date ___________**
